# Construction of Glycometabolism- and Hormone-Related lncRNA-Mediated Feedforward Loop Networks Reveals Global Patterns of lncRNAs and Drug Repurposing in Gestational Diabetes

**DOI:** 10.3389/fendo.2020.00093

**Published:** 2020-03-06

**Authors:** Xuelian Fu, Huifang Cong, Shuyu Zhao, Yan Li, Tianyi Liu, Yuhong Sun, Nan Lv

**Affiliations:** ^1^Department of Endocrinology, Second Affiliated Hospital of Harbin Medical University, Harbin, China; ^2^Department of Gynecology, Second Affiliated Hospital of Heilongjiang University of Chinese Medicine, Harbin, China; ^3^Third Ward of Obstetrics and Gynecology, Second Affiliated Hospital of Harbin Medical University, Harbin, China

**Keywords:** lncRNA-mediated feedforward loop, glycometabolism, hormone, gestational diabetes, drug repurposing, thyroid hormone

## Abstract

Gestational diabetes mellitus (GDM) is a condition associated with the onset of abnormal glucose tolerance during pregnancy. Long non-coding RNAs (lncRNAs), microRNAs (miRNAs), and genes can form lncRNA-mediated feedforward loops (lnc-FFLs), which are functional network motifs that regulate a wide range of biological processes and diseases. However, lnc-FFL network motifs have not been systematically investigated in GDM, and their role in the disease remains largely unknown. In the present study, a global lnc-FFL network was constructed and analyzed. Glycometabolism- and hormone-related lnc-FFL networks were extracted from the global network. An integrated algorithm was designed to identify dysregulated glycometabolism- and hormone-related lnc-FFLs in GDM. The patterns of dysregulated lnc-FFLs in GDM were complex. Moreover, there were strong associations between dysregulated glycometabolism- and hormone-related lnc-FFLs in GDM. Core modules were extracted from the dysregulated lnc-FFL networks in GDM and showed specific and essential functions. In addition, dysregulated lnc-FFLs could combine with ceRNAs and form more complex modules, which could play novel roles in GDM. Notably, we discovered that the dysregulated lnc-FFLs were enriched in the thyroid hormone signaling pathway. Some drug-repurposing candidates, such as hormonal drugs, could be identified based on lnc-FFLs in GDM. In summary, the present study highlighted the effect of dysregulated glycometabolism- and hormone-related lnc-FFLs in GDM and revealed their potential for the discovery of novel biomarkers and therapeutic targets for GDM.

## Introduction

Gestational diabetes mellitus (GDM), or glucose intolerance with first onset and recognition in pregnancy, is a common disease among pregnant women (1). Although the diagnostic criteria and the optimal protocol for detection and treatment of GDM are under debate, there is universal recognition that GDM can increase the risk for type 2 diabetes (2). Patients with GDM usually develop type 2 diabetes at a relatively younger age (<40 years) than women without GDM, have a higher risk of cardiovascular diseases, nonalcoholic fatty liver disease, as well as renal disease, and exhibit higher rates of early mortality (3, 4). Among the risk factors for GDM are pre-pregnancy weight gain and obesity, a family history of diabetes, an advanced maternal age, a poor diet, and low physical activity (5). While GDM is considered to stem from a diminished capacity of the pancreas to produce sufficient insulin and an impaired insulin action related to pregnancy, the detailed and global mechanism causing GDM remain uncertain.

Marked changes occur during maternal metabolism, and many of these are essential for healthy fetal growth and development. However, they may also lead to GDM and other diseases if there is a substantial deviation from the physiological gestational levels (6, 7). Pregnant women with GDM generally exhibit metabolic abnormalities, with alterations in lipid, glucose, and carbohydrate metabolism (8). Fetal exposure to hyperglycemia during a critical developmental period may have long-term effects on the fetus by creating a metabolic memory, also known as fetal programming (9). Certain hormones, such as progestogens, estrogens, and androgens, are essential for the successful establishment and maintenance of pregnancy and the proper development of the fetus. Hormone synthesis is generally abnormal in GDM patients (10). The thyroid hormone is an essential hormone for many biological processes and plays import roles in glucose metabolism and homeostasis. It has been suggested that thyroid hormone abnormalities play a role in the etiology of GDM (11). Although there are strong associations between glycometabolism, hormones, and GDM, the details of the mechanism are unknown.

Recently, genetic studies have focused on long non-coding RNAs (lncRNAs), which are defined as non-coding RNAs more than 200 nucleotides in length (12). lncRNAs are known to play a vital role in cellular development and many biological process and diseases (13–15). A previous study has suggested that circulating lncRNA could serve as a fingerprint for GDM, and it is associated with a macrosomia risk (16). The expression of lncRNA MALAT1 could offer a novel biomarker for predicting GDM (17). Genes, microRNAs (miRNAs), and their shared target lncRNAs can form lncRNA-associated feedforward loops (lnc-FFLs), in which genes and miRNAs co-ordinate to regulate lncRNA expression (18). The regulatory units within an lnc-FFL network are comprised of a gene, an miRNA, and their shared target lncRNAs. FFLs participate in many biological processes, including cell development and differentiation, and can cause disease (19, 20). However, the functions of lnc-FFL in GDM, and especially the relationship between lnc-FFL, glycometabolism, and hormones, are unclear.

In the present study, a global lnc-FFL network was constructed and analyzed. Glycometabolism- and hormone-related lnc-FFL networks were extracted, and it was shown that lncRNAs play essential roles in these two networks. An integrated computational approach was designed to identify dysregulated glycometabolism- and hormone-related lnc-FFLs in GDM patients. The patterns of dysregulated glycometabolism- and hormone-related lnc-FFLs in GDM are complex. There are strong associations between dysregulated glycometabolism- and hormone-related lnc-FFLs in GDM. Some core modules were discovered and extracted from dysregulated lnc-FFLs in GDM. These dysregulated lnc-FFLs showed specific and essential functions. Furthermore, the dysregulated lnc-FFLs and ceRNAs could form more complex modules that play additional roles in GDM. In particular, we discovered that these dysregulated lnc-FFLs focused on the thyroid hormone signaling pathway. Some drug repurposing candidates were also identified based on lnc-FFL in GDM. Collectively, the present study highlighted the effect of dysregulated glycometabolism- and hormone-related lnc-FFLs in GDM and revealed their potential as novel biomarkers and treatment targets in GDM.

## Materials and Methods

### Construction of a Global Experimentally Validated lnc-FFL Network

Data for experimentally validated gene–miRNA interactions were obtained from miRTarBase 7.0, which is a public database that contains more than 360,000 miRNA–target interactions (21). For the current study, we only extracted gene–miRNA interactions supported by strong experimental evidence. All experimentally validated gene–lncRNA and miRNA–lncRNA interactions were obtained from the RAID 2.0 database (22). RAID contains experimentally verified and computationally predicted RNA–RNA interactions. Only gene–lncRNA and miRNA–lncRNA interactions supported by strong experimental evidence were extracted. Based on these experimentally verified interactions, an lnc-FFL was identified whereby a specific gene would regulate an lncRNA and an miRNA, while the miRNA would also regulate the lncRNA. Then, based on all of the lnc-FFLs identified above, the global lnc-FFL network was constructed using cytoscape (http://www.cytoscape.org/). The analysis of the topological features of the network was also performed in cytoscape.

### Characteristics of GDM and NGT Samples

All the GDM and normal glucose-tolerant (NGT) samples were obtained from a previous study (23). All the subjects received 5-point 75-g oral glucose tolerance test after fasting for one night. Blood glucose and insulin were measured at 0, 30, 60, 90, and 120 min. A Bedside glucose analyzer (YSI, Yellow Springs, OH, USA) and ADVIA Centaur XP (Siemens Healthcare GmbH, Erlangen, Germany) were used to measure blood glucose and plasma insulin. NGT samples were defined as fasting glucose ≤ 5.11 mmol/L, 1-h glucose ≤ 10.00 mmol/L, and 2-h glucose ≤ 8.50 mmol/L. GDM patients were defined as the fasting glucose beyond the above limits. GDM was diagnosed based on the International Association of diabetes and pregnancy study groups in 2010 recommendations for the diagnosis and classification of hyperglycemia during pregnancy. At last, the present study contained eight GDM and NGT pregnant women who were matched based on their body-mass-index and age. The anthropometric and metabolic characteristics of GDM and NGT samples were summarized in Table S1.

### Collection of High-Throughput Expression Profiles of lncRNAs, miRNAs, and Genes for GDM

The high-throughput expression profiles of lncRNAs, miRNAs, and genes for GDM and normal control samples were obtained from the Gene Expression Omnibus database (www.ncbi.nlm.nih.gov/geo). Whole blood was collected from these 16 subjects in PAXgene Blood RNA Tubes (PreanalytiX) after overnight fasting. Next, total RNA was isolated using the PAXgene Blood miRNA kit (PreanalytiX) according to the manufacturer's specifications. Paired data were adjusted for mid-pregnancy weight gain and pregnancy week (GSE92772). The Illumina HiSeq 2500 sequencer was used to obtain the blood cell RNA-sequencing data from the above 16 samples. Adapter trimming was performed using trim_galore/cutadapt v 0.4.0 with standard settings for Illumina reads. Read mapping against the hg38 genome was performed using STAR aligner v 2.4.1d with standard settings. The expression and detailed information about the dataset can be obtained from the GSE92772 database (https://www.ncbi.nlm.nih.gov/geo/query/acc.cgi?acc=GSE92772).

### Collection of Glycometabolism- and Hormone-Related Genes

All glycometabolism- and hormone-related genes were obtained from AmiGO 2 version: 2.4.26 in Homo sapiens species (24). Finally, we collected 1,845 glycometabolism-related genes and 1,552 hormone-related genes.

### Identification of Dysregulated Glycometabolism- and Hormone-Related lnc-FFLs in GDM

We designed a comprehensive computational approach to identify significantly dysregulated lnc-FFLs for GDM using global lnc-FFL network and expression profile data. This integrated approach focused on whole change of the L-FFL rather than a single molecule. Firstly, we performed a Student *t*-test for each lnc-FFL to evaluate differences in lncRNA, miRNA, and gene expression levels between GDM and NGT patients. The resulting *p*-values from the *t*-tests were used in consequent analyses. Secondly, we calculated Pearson Correlation Coefficients (PCCs) for each gene–lncRNA, gene–miRNA, and miRNA–lncRNA interaction in the GDM and NGT samples. For each interaction, the absolute difference between PCC values from the GDM and NGT samples was defined as the difference level for that specific interaction. Thirdly, for each lnc-FFL, two comprehensive scores (CSs) containing differential expression *P*-values (CS_dif_) and PCCs (CS_PCC_) were defined:

(1) $$\begin{array}{l} \text{C}{\text{S}}_{\text{dif}}={\text{P}}_{\text{lncRNA}}*{\text{P}}_{\text{miRNA}}*{\text{P}}_{\text{gene}} \end{array}$$

(2) $$\begin{array}{ll} \begin{array}{l} {\text{CS}}_{\text{PCC}}=|({\text{GDM}}_{\text{lnc}-\text{miR}}{-\text{ NGT}}_{\text{lnc}-\text{miR}})*({\text{GDM}}_{\text{lnc}-\text{gene}}-\\ \;{\text{NGT}}_{\text{lnc}-\text{gne}})*({\text{GDM}}_{\text{miR}-\text{gene}}{-\text{ NGT}}_{\text{miR}-\text{gene}})| \end{array} & \;\left(2\right) \end{array}$$

where P_lncRNA_, P_miRNA_, and P_gene_ denote the *p*-values for the differential lncRNA, miRNA, and gene expression, respectively, for each lnc-FFL. CS_dif_ represents the integrated differential lncRNA, miRNA, and gene expression levels between the GDM and NGT samples. GDM_lnc−miR_, GDM_lnc−gene_, and GDM_miR−gene_ represent the PCC values of lncRNA-miRNA, lncRNA-gene, and gene-miRNA interactions, respectively, in GDM patients. Similarly, NGT_lnc−miR_, NGT_lnc−gene_, and NGT_miR−gene_, represent the PCC values of lncRNA–miRNA, lncRNA–gene, and gene–miRNA interactions, respectively, in NGT samples. CS_PCC_ represents the difference in the co-expression levels of an lnc-FFL between GDM and NGT. Fourthly, we used the values of CS_dif_ and CS_PCC_ to rank all lnc-FFLs based on an equal weighted ranking method, and each lnc-FFL was then given a final comprehensive score. Finally, we performed 1,000 random permutations of the sample labels in the lncRNA, miRNA, and gene expression profiles. Then, the significant lnc-FFLs were obtained by comparing their final comprehensive score with their permutation score (*p* < 0.05).

### Identification of Key and Core Modules From the Dysregulated lnc-FFL Network Associated With GDM

Key modules containing the node with the highest degree and its neighbors were extracted. Core modules were extracted from the dysregulated lnc-FFL network associated with GDM using the package ClusterOne in cytoscape with default parameters (http://apps.cytoscape.org/apps/ClusterONE). ClusterONE is a package that clusters a given network based on its topology in order to identify densely connected regions.

### Functional Enrichment Analysis for Dysregulated lnc-FFLs in GDM Patients

In order to describe the functions of dysregulated lnc-FFLs in GDM, we used the DIANA-miRPath v3.0 software to analyze miRNA functions, thus inferring the functions of the corresponding lnc-FFLs and lncRNAs (25). Several significant GO terms and KEGG pathways were identified.

### Analysis of Drug Repurposing Candidates Based on GDM lnc-FFLs

DrugBank was used to obtain information about the relation between drugs and genes in dysregulated lnc-FFLs (26). The SM2miR database was used to obtain information about the relation between drugs and miRNAs in dysregulated lnc-FFLs (27).

## Results

### Construction and Analysis of Glycometabolism- and Hormone-Related lnc-FFL Networks

**Figure 1:**
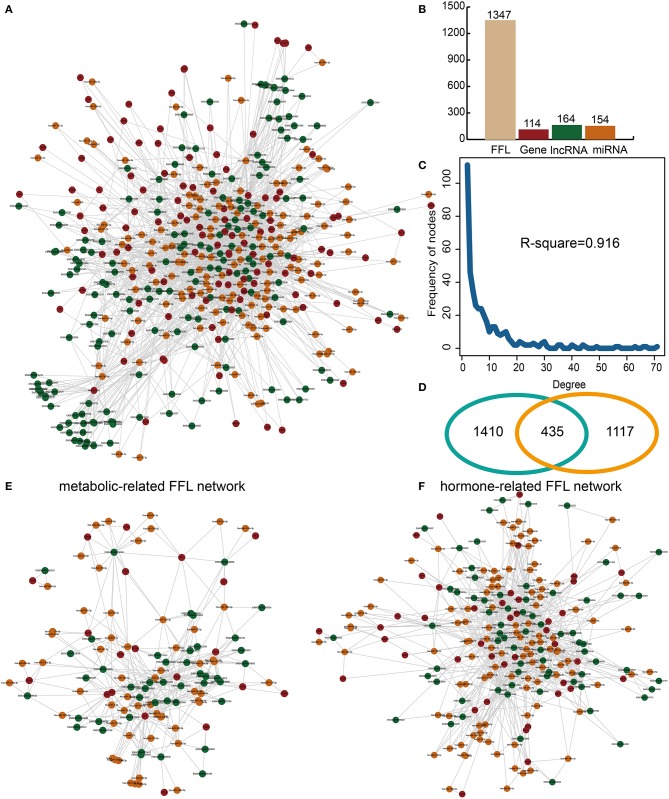
Construction and analysis of glycometabolism- and hormone-related lnc-FFL networks. **(A)** A global lnc-FFL network based on experimentally verified interaction data. lncRNAs, miRNAs, and genes are colored in green, yellow, and red, respectively. **(B)** Bar plot, showing the number of lncRNAs, miRNAs, and genes in the global lnc-FFLs network. **(C)** The degree distribution of the global lnc-FFL network. **(D)** Venn diagram, showing the intersection between glycometabolism- and hormone-related genes. **(E)** The glycometabolism-related lnc-FFL network. **(F)** The hormone-related lnc-FFL network.

lnc-FFL is defined as a regulatory motif whereby a gene regulates an lncRNA and an miRNA while the miRNA also regulates the lncRNA. Multiple lnc-FFLs could form an lnc-FFL network. In our study, a global lnc-FFL network was constructed based on data describing experimentally verified gene–lncRNA, gene–miRNA, and miRNA–lncRNA interactions (Figure 1A). The global lnc-FFL network contained 1,347 lnc-FFLs, 114 coding genes, 164 lncRNAs, and 154 miRNAs (Figure 1B). We discovered that the global lnc-FFL network approximates a scale-free network (*R*-square = 0.916), which is a major topological feature of biology network (Figure 1C). This indicates that the global lnc-FFL network provides an effective context for identifying specific lnc-FFLs of GDM. Previous study suggested that glycometabolism and hormones are two highly influential factors in GDM (8, 10). We obtained 1,845 glycometabolism- and 1,552 hormone-related genes. The intersection between glycometabolism- and hormone-related genes contained 435 members, suggesting that there is a link between these two factors (Figure 1D). In order to describe the associations between glycometabolism- and hormone-related lnc-FFLs in GDM, we extracted glycometabolism- and hormone-related lnc-FFL subnetworks from the global lnc-FFL network (Figures 1E,F). The glycometabolism-related lnc-FFL network included 229 lnc-FFLs, 20 coding genes, 44 lncRNAs, and 58 miRNAs. The hormone-related lnc-FFL network included 530 lnc-FFLs, 42 coding genes, 69 lncRNAs, and 111 miRNAs. These lncRNAs and miRNAs maybe play important roles in the glycometabolism- and hormone-related lnc-FFL network. All these results indicate that lnc-FFLs are vital motifs in the biology of GDM.

### Some Glycometabolism- and Hormone-Related lnc-FFLs Are Dysregulated in GDM Patients

**Figure 2:**
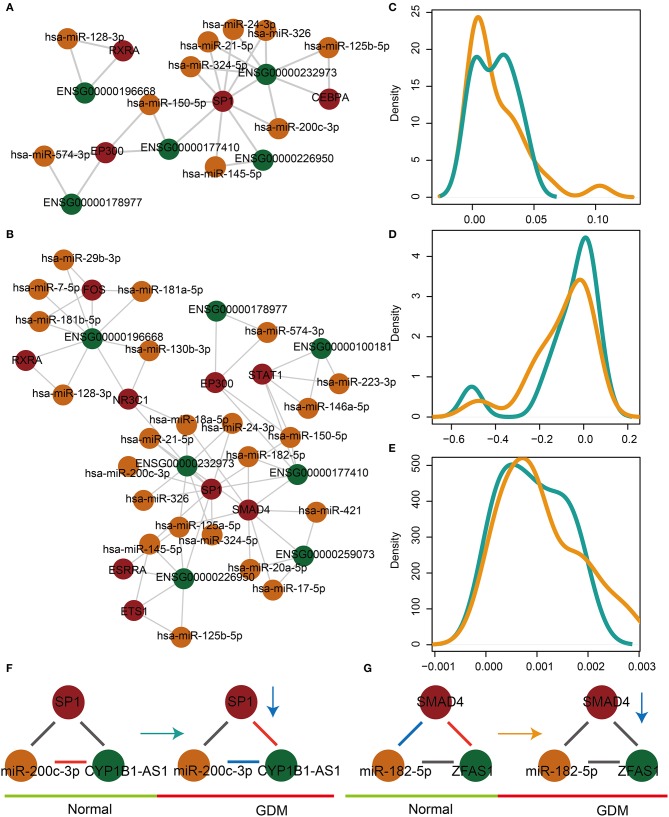
The dysregulated glycometabolism- and hormone-related lnc-FFL networks. **(A)** The dysregulated glycometabolism-related lnc-FFL network. **(B)** The dysregulated hormone-related lnc-FFL network. **(C–E)** Density distribution curves of CS_dif_, CS_PCC_, and final score, respectively, in dysregulated glycometabolism- and hormone-related lnc-FFL networks. **(F,G)** Two examples showing dysregulated patterns of lnc-FFLs in GDM.

A comprehensive algorithm was developed to identify significantly dysregulated lnc-FFLs in the GDM-associated glycometabolism- and hormone-related lnc-FFL using the global lnc-FFL network and lncRNA, miRNA, and gene expression profiles. We identified 11 glycometabolism- and 29 hormone-related lnc-FFLs that were dysregulated in GDM (Figures 2A,B). Then, two comprehensive scores based on CS_dif_ and CS_PCC_ as well as a final score were used to characterize the networks. All three scores showed a similar distribution in both glycometabolism- or hormone-related lnc-FFL networks (Figures 2C–E). Moreover, CS_dif_ and the final score approximated a unimodal distribution, whereas CS_PCC_ followed a bimodal-peaks distribution. Notably, we discovered that the dysregulated patterns of lnc-FFLs are multiple and complex. For example, in the dysregulated glycometabolism-related lnc-FFL SP1/miR-200c-3p/CYP1B1-AS1, the interaction between miR-200c-3p and CYP1B1 changed from a positive correlation to a negative correlation (Figure 2F), whereas the interaction between SP1 and CYP1B1 changed from no correlation to a positive correlation. The SP1 gene is downregulated in GDM patients, yet no change in the interaction between SP1 and miR-200c-3p was observed. In another dysregulated hormone-related lnc-FFL SMAD4/miR-185-5p/ZFAS1, the interaction between SMAD4 and miR-182-5p changed from a negative correlation to no correlation (Figure 2G), and the interaction between SMAD4 and ZFAS1 changed from a positive correlation to no correlation. The respective coding gene is downregulated in GDM patients. These results indicate that only local changes in the lnc-FFL networks can contribute to dysregulation in GDM. The resulting patterns in the dysregulated lnc-FFLs are multiple and complex.

### The Dysregulated Glycometabolism- and Hormone-Related lnc-FFLs Show Strong Associations in GDM Patients

**Figure 3:**
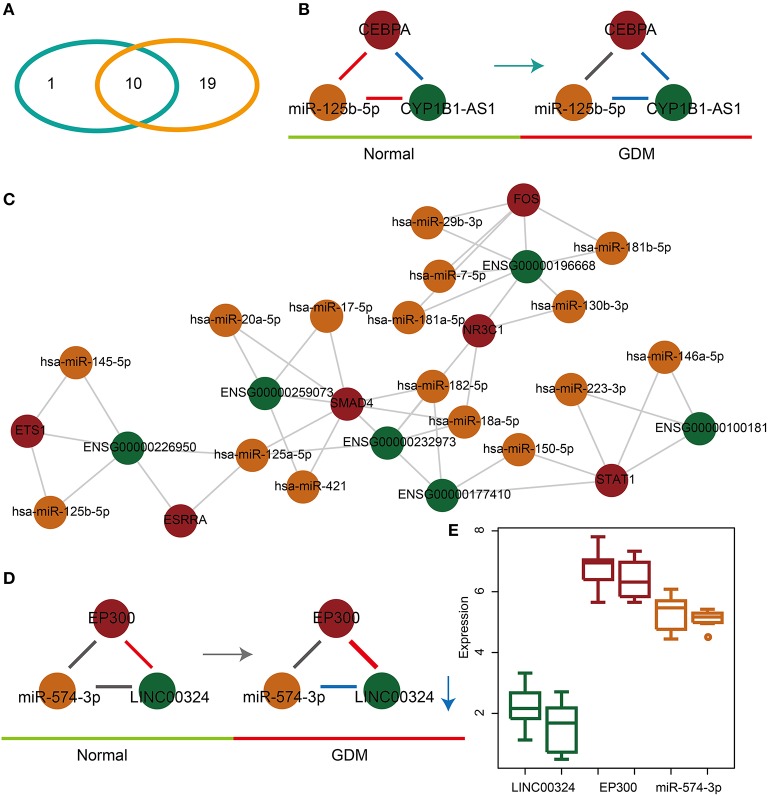
Common and specific features of dysregulated glycometabolism- and hormone-related lnc-FFLs. **(A)** Venn diagram showing the intersection between dysregulated glycometabolism- and hormone-related lnc-FFLs. **(B)** The glycometabolism-related lnc-FFL network in GDM. **(C)** The hormone-related dysregulated lnc-FFL network in GDM. **(D)** An example showing the common dysregulated lnc-FFLs in GDM. **(E)** lncRNA, miRNA, and gene expression levels in a common dysregulated lnc-FFL.

In order to explore the associations between dysregulated glycometabolism- and hormone-related lnc-FFLs, we further analyzed both the common and unique dysregulated glycometabolism- and hormone-related lnc-FFLs in GDM. We identified 10 common glycometabolism- and hormone-related dysregulated lnc-FFLs in GDM (Figure 3A). An lnc-FFL dysregulated only in the glycometabolism-related networks is the CEBPA/miR-125b-5p/CYB1B1-AS1 module (Figure 3B). In this dysregulated lnc-FFL, all interactions between gene, miRNA, and lncRNA are perturbed. Similarly, we also constructed a specific hormone-related dysregulated lnc-FFL network containing 19 lnc-FFLs, six coding genes, six lncRNAs, and 16 lncRNAs (Figure 3C). These lnc-FFLs are all specific for the hormone-related dysregulated lnc-FFL networks associated with GDM. In the specific hormone-related dysregulated lnc-FFL EP300/miR-574-3p/LINC00324, only the interaction of miR-574-3p and LINC00324 appeared to change from no correlation to a negative correlation (Figure 3D). The interaction between EP300 and LINC00324 was stronger in GDM. We also discovered that the changes in gene, miRNA, and lncRNA expression levels are weak (Figure 3E). However, the interactions between these three molecules show major changes; therefore, they may be important for the development of GDM.

### Certain Core Modules Exhibit Specific Functions and lnc-FFLs Could Form More Complex Modules With ceRNAs

**Figure 4:**
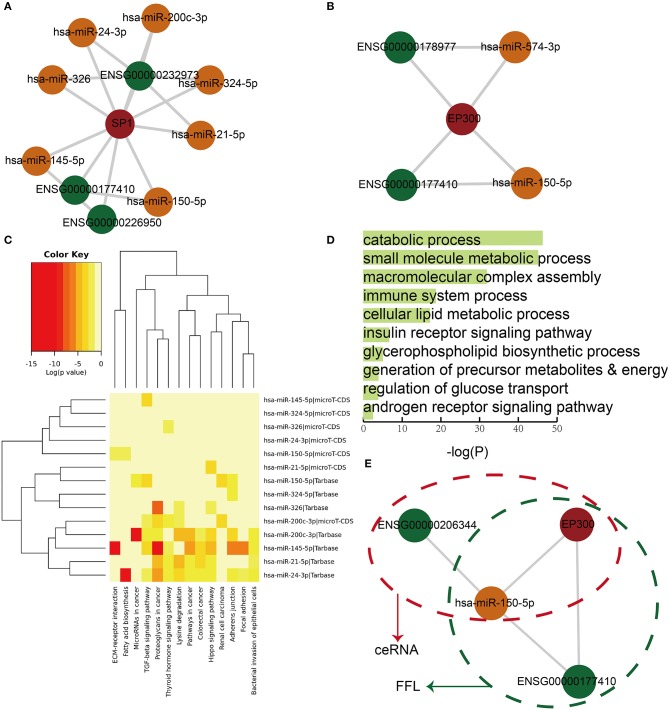
Core modules extracted from common dysregulated lnc-FFL networks, showing specific functions. **(A)** A key module that contains the node with the highest degree and its neighbors. **(B)** A core module extracted from dysregulated lnc-FFL networks associated with GDM. **(C)** Heat map showing the *p*-values of pathway enrichment analysis with red representing more significant *p*-values. **(D)** Bar plot showing the *p*-values of significantly enriched GO terms. **(E)** An example, showing that ceRNAs and dysregulated lnc-FFLs combine to form more complex modules.

In order to further characterize the roles of dysregulated lnc-FFLs in GDM, we analyzed the lnc-FFL modules extracted from common glycometabolism- and hormone-related dysregulated lnc-FFLs. First, we extracted a key module, which contained the SP1 gene exhibiting the highest degree and all its neighbors (Figure 4A). This key module contained three lncRNAs and seven miRNAs. In addition, we identified a core module, which showed a closer network structure (Figure 4B). This core module consisted of one coding gene, two lncRNAs, two miRNAs, and five molecules from two lnc-FFLs: EP300/LINC00324/miR-150-5p and EP300/ZFAS1/miR-574-3p. We discovered that miRNAs play vital roles in these two modules; therefore, we analyzed the functions of these miRNAs to better understand the function of the whole module. We found that these miRNAs are enriched in some key pathways, such as the thyroid hormone signaling pathway, the fatty acid biosynthesis pathway, and the lysine degradation pathway (Figure 4C). They are also enriched in some GO terms, such as the catabolic process, the small molecule metabolic process, the insulin receptor signaling pathway, and the androgen receptor signaling pathway (Figure 4D). Almost all of the enrichment pathways and GO terms are related with metabolic and hormone regulation processes. Importantly, we discovered that some dysregulated lnc-FFLs can combine with dysregulated ceRNAs to form more complex modules that exhibit distinct functions. For example, lnc-FFL EP300/ZFAS1/miR-150-5p and ceRNA EP300/HCG27/miR-150-5p form a complex module by sharing EP300 and miR-150-5p (Figure 4E). Our results indicated that some modules extracted from dysregulated lnc-FFL networks show specific functions and could combine with other motifs to gain new functionalities.

### Identification of Drug Repurposing Candidates for GDM Based on Dysregulated lnc-FFLs

**Figure 5:**
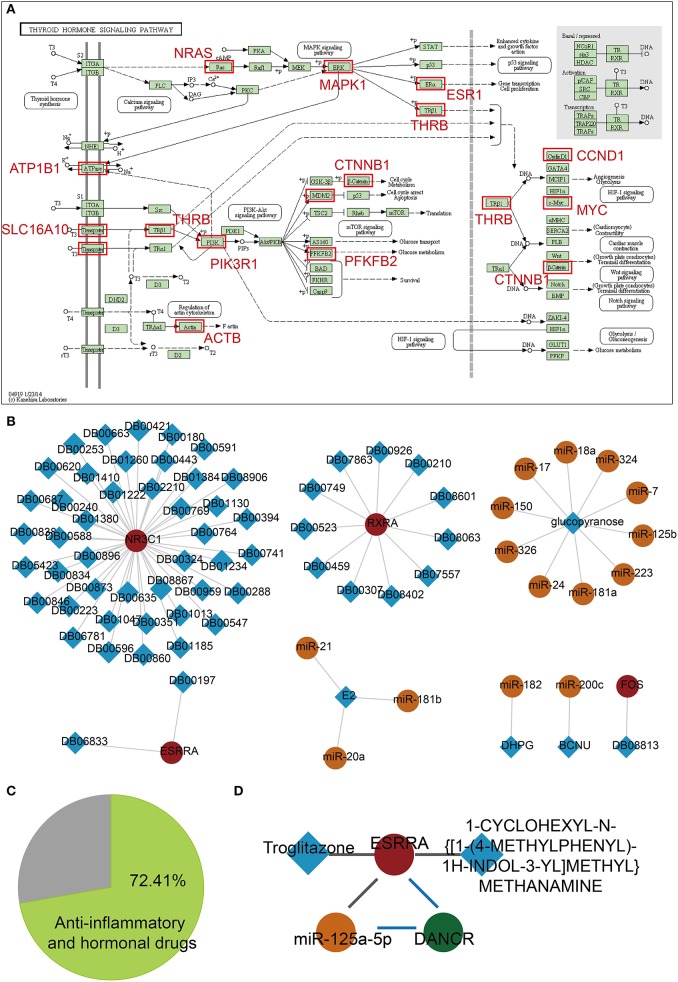
Candidate drug repurposing based on dysregulated lnc-FFLs associated with GDM. **(A)** The thyroid hormone pathway, and enriched target genes of miRNAs in dysregulated lnc-FFLs associated with GDM. **(B)** Drug-related dysregulated lnc-FFL networks. The square represents drugs and the circle represents genes, lncRNAs, and miRNAs. **(C)** Pie chart showing the percent of anti-inflammatory and hormonal drugs. **(D)** Candidate drug repurposing of the ESPRA-related lnc-FFL.

Based on functional analyses, we found a key pathway, namely, the thyroid hormone signaling pathway, which is related with dysregulated lnc-FFLs in GDM (Figure 5A). The thyroid gland and its metabolism have a considerable physiological impact during pregnancy (28). Thyroid dysfunction is considered to play a vital role in the etiology of GDM because the thyroid hormone plays an important role in glucose metabolism and homeostasis (11). We discovered that some of the target genes of miRNAs in dysregulated lnc-FFLs are enriched in the thyroid hormone signaling pathway. For example, PFKFB2 is a key gene in the thyroid hormone signaling pathway and directly regulates glucose metabolism. Finally, we considered the possibility that dysregulated lnc-FFLs could contribute to the identification of drug repurposing candidates for GDM. For this purpose, we constructed a drug-related dysregulated lnc-FFL network based on genes and miRNAs in dysregulated lnc-FFLs networks associated with GDM (Figure 5B). This drug-related network contained 4 genes, 15 miRNAs, and 62 drugs, 72.41% of which are anti-inflammatory and hormonal drugs, such as prednisone, hydrocortisone, paramethasone, and ciclesonide (Figure 5C). In particular, we discovered a candidate drug named troglitazone, which was introduced as an anti-diabetic drug but was subsequently withdrawn from clinical use due to severe hepatotoxicity (29). We found that the ESRRA (steroid hormone receptor ERR1) gene is a target of troglitazone and forms a dysregulated lnc-FFL with miR-125b-5p and CYP1B1-AS1 (Figure 5D). Another drug candidate was 1-CYCLOHEXYL-N-{[1-(4-METHYLPHENYL)-1H-INDOL-3-YL]METHYL}METHANAMINE, whose function and mechanism of action is uncertain. These results indicated that some anti-inflammatory and hormonal drugs can be considered candidate drugs for GDM.

## Discussion

lncRNAs, miRNAs, and coding genes can form many types of motifs that play important roles in many biological processes and a large number of diseases. At present, there are many studies focusing on ceRNAs and FFLs. Wu et al. constructed a comprehensive ceRNA network and identified key molecules in diabetic retinopathy (30). Ling et al. also validated some ceRNA motifs in diabetic retinopathy (31). FFLs are network motifs, defined by a three-gene pattern, which are composed of two input transcription factors, one of which regulates the other, and both jointly regulate a target gene (32). In our study, we constructed an lncRNA-mediated FFL, composed of one gene, one lncRNA, and one miRNA, whereby the gene regulates the miRNA, and both the gene and the miRNA regulate the lncRNA. Such lnc-FFLs play important roles in many diseases, including cancer (18, 33, 34). In the present study, we developed an integrated algorithm to identify dysregulated lnc-FFLs associated with GDM. We attempted to define the functions of such dysregulated lnc-FFLs in GDM based on functional and drug repurposing analyses. Our results indicated that dysregulated lnc-FFLs play important roles in GDM.

GDM is a disease characterized by metabolic dysfunction that manifests during pregnancy. Typically, alterations in lipid, glucose, and carbohydrate metabolism occur in pregnant women with GDM (35). In the present study, we focused on glycometabolism, which includes fucosylation, lipid glycosylation, macromolecule glycosylation, and mannosylation. A previous study suggested that, during normal pregnancy, pathways involved in the biosynthesis of steroid hormones are tightly regulated (36). The roles and underlying mechanisms of glycometabolism and hormones in GDM are unclear. In our study, we identified glycometabolism- and hormone-related dysregulated lnc-FFLs in GDM. We discovered a significant intersection between glycometabolism- and hormone-related dysregulated lnc-FFLs in GDM, suggesting that glycometabolism and hormone-related lnc-FFLs may act jointly to contribute to the occurrence and development of GDM. However, there were more hormone-related lnc-FFLs than glycometabolism-related lnc-FFLs associated with GDM. Thus, hormonal disorders may play more complex and important roles in GDM.

Pregnancy alters normal thyroid function, and severe maternal hypothyroidism has been associated with an increased risk for GDM (37). The levels of blood glucose and thyroid function of pregnant women depend on many hormones, including estrogen, thyroid-binding globulin, human chorionic gonadotropin, placental lactogen, cortisol, and placental insulin enzyme (38). An increasing number of studies suggests that there is a strong association between thyroid diseases and GDM (39). In our analyses, we found that the thyroid hormone signaling pathway is a key pathway in which the target genes of miRNAs in dysregulated lnc-FFLs are enriched. The thyroid hormone signaling pathway contains many important carbohydrate metabolism-related subpathways or processes, such as glucose transport and glucose metabolism. Our results, based on the analysis of dysregulated lnc-FFLs, demonstrated that there is a strong association between thyroid function and GDM.

Another important network motif that contributes to the development of many diseases involves ceRNA (40). In our analyses, we found 11 ceRNAs sharing common genes, miRNAs, or lncRNAs with dysregulated lnc-FFLs in GDM. The dysregulated lnc-FFL and ceRNA motifs might exhibit complex regulatory functions and crosstalk in GDM. Our results suggested that many kinds of network motifs influence the development of GDM. Moreover, these networks are not independent of each other, and they generally form more complex motifs that may assume new roles in GDM. Future studies will focus on investigating a higher number of GDM samples in order to validate the accuracy and stability of the method presented in the current study. The integrated method could provide assistance for exploring the roles of lncRNAs in GDM by their interacted genes and miRNAs. The comprehensive approach by integrating expression profiles of gene, miRNA, and lncRNA could identify better and more global biomarkers for GDM than a single type of molecule. However, the size of the samples, which detected the expression of lncRNAs, miRNAs, and genes at the same time, was not very large. In future work, more GDM and NGT samples should be used for validating this method. Although we were only able to validate the drug repurposing candidates in a small sample size, these served as proof of principles for using the lnc-FFLs in expression and interactions panels to gain insight into precision medicine. With the emergence of the pharmacogenomics data of standardly designed GDM precision medicine, we should be able to determine the performance of lnc-FFLs in GDM patients in short future.

In summary, in the present study, a global lnc-FFL network was constructed. An integrated algorithm was designed and significantly dysregulated glycometabolism- and hormone-related lnc-FFLs were identified in connection with GDM. The patterns of the dysregulated lnc-FFLs associated with GDM are multiple and complex. The identified glycometabolism- and hormone-related lnc-FFLs showed strong association with GDM. Core modules with specific functions in GDM could be extracted from the dysregulated lnc-FFLs networks. In addition, dysregulated lnc-FFLs could form more complex modules through combination with ceRNAs. Drug repurposing candidates, such as hormone-related drugs, were identified based on lnc-FFLs associated with GDM. Our study provides insight for the identification of novel biomarkers and drug candidates for GDM.

## Data Availability Statement

All data generated or analyzed during this study are included in this published article.

## Author Contributions

NL and XF conceived and designed the experiments. HC, SZ, YL, and TL analyzed the data. NL and YS wrote the manuscript.

### Conflict of Interest

The authors declare that the research was conducted in the absence of any commercial or financial relationships that could be construed as a potential conflict of interest.
