# Supplementary material for: Construction of Glycometabolism- and Hormone-Related lncRNA-Mediated Feedforward Loop Networks Reveals Global Patterns of lncRNAs and Drug Repurposing in Gestational Diabetes
Source: Front Endocrinol (Lausanne). 2020 Mar 6;11:93. doi: 10.3389/fendo.2020.00093 (PMC7068675; doi:10.3389/fendo.2020.00093)
Supplement: Supplementary file 1 [file Table_1.DOC]

Table S1. Anthropometric and metabolic characteristics of GDM and NGT samples.

|  | GDM | NGT | Significance |
| --- | --- | --- | --- |
| Number | 8 | 8 | NA |
| Age (years) | 32± 3 | 33± 5 | 0.7 |
| Body mass index (kg/m^2^) | 29.1± 6.1 | 28.1± 5.4 | 0.7 |
| Pregnancy week (weeks) | 25.9± 1.7 | 23.0± 9.5 | 0.4 |
| Fasting glucose (mmol/L) | 4.89± 0.62 | 4.44± 0.27 | 0.09 |
| 1 hour glucose (mmol/L) | 10.90± 0.82 | 7.76± 1.21 | 0.00013 |
| 2 hour glucose (mmol/L) | 7.52± 1.44 | 6.32± 1.31 | 0.1 |
| Fasting insulin (pmol/L) | 126.38± 53.70 | 59.88± 28.30 | 0.01 |
| Insulin sensitivity index (10^19^L^2^/mol^2^) | 5.0± 2.0 | 13.6± 6.8 | 0.03 |
| Maternal weight gain (kg) | 11.27± 9.64 | 6.53± 3.38 | 0.2 |
| Family history of parental diabetes | Paternal= 1 Maternal= 0 | Paternal= 0 Maternal= 0 | 0.2 |

**Table Legend:** Table S1 shows anthropometric and metabolic characteristics of GDM and NGT samples. All data are given as absolute numbers or means ± SD. Significant differences calculated in a Student's t-test or Chi-square-test (family history of diabetes).
